# The highly flexible disordered regions of the SARS-CoV-2 nucleocapsid N protein within the 1–248 residue construct: sequence-specific resonance assignments through NMR

**DOI:** 10.1007/s12104-021-10009-8

**Published:** 2021-03-03

**Authors:** Marco Schiavina, Letizia Pontoriero, Vladimir N. Uversky, Isabella C. Felli, Roberta Pierattelli

**Affiliations:** 1 Magnetic Resonance Center – CERM, University of Florence, Via Luigi Sacconi 6, 50019 Sesto Fiorentino, FI Italy; 2 Department of Chemistry “Ugo Schiff”, University of Florence, Via della Lastruccia 3-13, 50019 Sesto Fiorentino, FI Italy; 3 Department of Molecular Medicine and USF Health Byrd Alzheimer’s Research Institute, Morsani College of Medicine, University of South Florida, Tampa, FL 33612 USA

**Keywords:** SARS-CoV-2, Covid-19, Nucleocapsid protein, NMR spectroscopy, ^13^C detection, IDPs

## Abstract

The nucleocapsid protein N from SARS-CoV-2 is one of the most highly expressed proteins by the virus and plays a number of important roles in the transcription and assembly of the virion within the infected host cell. It is expected to be characterized by a highly dynamic and heterogeneous structure as can be inferred by bioinformatics analyses as well as from the data available for the homologous protein from SARS-CoV. The two globular domains of the protein (NTD and CTD) have been investigated while no high-resolution information is available yet for the flexible regions of the protein. We focus here on the 1–248 construct which comprises two disordered fragments (IDR1 and IDR2) in addition to the N-terminal globular domain (NTD) and report the sequence-specific assignment of the two disordered regions, a step forward towards the complete characterization of the whole protein.

## Biological context

Coronaviruses (CoVs) are relatively large viruses containing a single-stranded positive-sense RNA genome encapsulated within a membrane envelope (Cui et al. 2019). There are four classes of CoVs, called α, β, γ, and δ, with the class β-coronavirus including CoVs that can infect humans, such as the severe acute respiratory syndrome virus (SARS-CoV), the Middle East respiratory syndrome virus (MERS-CoV), and the COVID-19 causative agent SARS-CoV-2 (Masters 2006; Surjit and Lal 2008). Similar to SARS-CoV and MERS-CoV, SARS-CoV-2 attacks the lower respiratory system causing viral pneumonia, but it may also affect the gastrointestinal system, heart, kidney, liver, and central nervous system leading to multiple organ failure (Huang et al. 2020; Wang et al. 2020). The severe rate of this virus spread, based on its unexpectedly high infectivity, demands rapid action towards both the development of a vaccine and potent viral inhibitors to weaken or eliminate major life-threat symptoms.

**Fig. 1:**
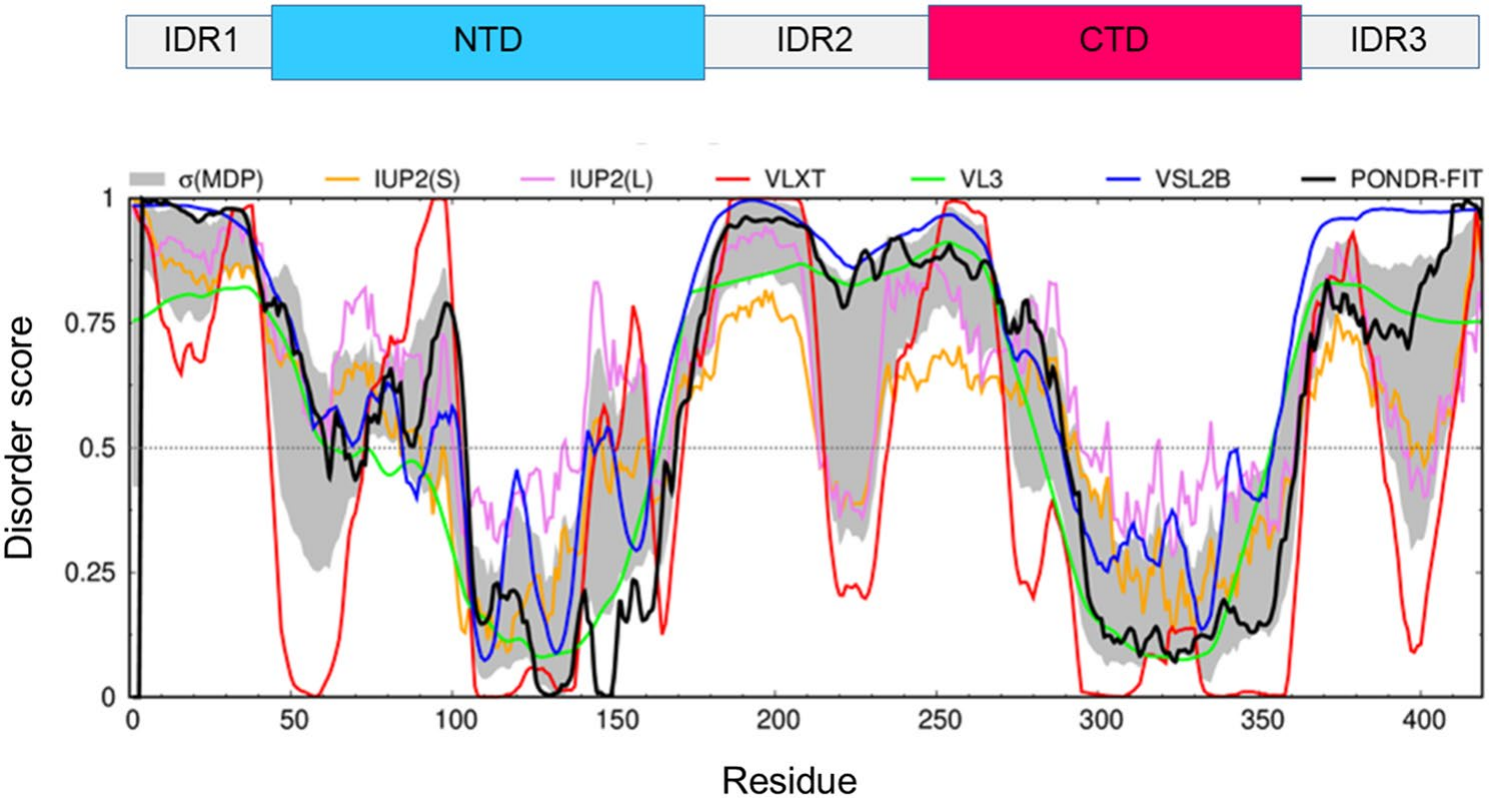
Bioinformatics analysis of the intrinsic disorder predisposition of the SARS-CoV-2 nucleocapsid N protein obtained using IUPred short (golden line), IUPred long (purple line), PONDR® VLXT (red line), PONDR® VL3 (green line), PONDR® VSL2B (blue line), PONDR® FIT (black line). The gray shadow region signifies the error distribution σ(MDP) around the mean disorder profile calculated by averaging of the disorder profiles of individual predictors. Protein regions with a disordered score consistently larger than 0.5 are considered disordered, whereas regions with disorder scores between 0.2 and 0.5 are considered as flexible. Over the plot, the domain organization used in the text is reported

The SARS-CoV-2 nucleocapsid protein N is a structurally heterogeneous, 419 amino-acid-long, multidomain RNA-binding protein that is found inside the viral envelope (Fig. 1). This protein, as already established for its SARS-CoV homologue, stabilizes viral RNA by forming a ribonucleoprotein complex (RNP) and plays a fundamental role in the transcription and assembly of the virion once the host cell is infected (Chang et al. 2009, 2014). The self-association of the N protein is also responsible for the formation of a shell, the capsid, which protects the genetic material from external agents. The N protein includes two functional domains known as N- and C-terminal domains, or NTD and CTD respectively, that are responsible for RNA binding (NTD) and homo-dimerization (CTD) (Chang et al. 2006). Bioinformatics analysis predicts the presence of three long intrinsically disordered regions in the polypeptide chain as reported in Fig. 1 (Giri et al. 2020). These regions are believed to be responsible for an intricate mechanism that leads to the regulation of the formation of the RNP complex. They are also engaged in many interactions with other viral proteins or host proteins, as was already demonstrated for the homologous nucleocapsid protein of the CoV that causes SARS (Chang et al. 2014; Giri et al. 2020). To date there is no structural and dynamic information with atomic resolution for the entire N protein due to its highly disordered nature. The structures of the globular NTD and CTD domains have been determined (Kang et al. 2020; Peng et al. 2020; Dinesh et al. 2020). However, there is no atomic resolution information on the disordered parts of this protein. On the other hand, the role of disorder is not accidental and is very relevant for the modulation of the mechanisms leading to the infection (Goh et al. 2012, 2013). In addition, the N proteins of the different variants of CoVs seem to be genetically stable (Giri et al. 2020), which makes them excellent candidates for developing antiviral therapies that have not been explored to date.

In this frame, we provide here the backbone assignment of the two disordered regions flanking the NTD, the N-terminal IDR1 and the serine-rich disordered region IDR2, in the 1–248 residue construct (IDR1-NTD-IDR2). These data will contribute to the efforts of the research consortium covid19-nmr (www.covid19-nmr.de) enabling follow-up applications, such as residue-resolved drug screening and interaction mapping.

## Methods and experiments

### Construct design

This study uses the SARS-CoV-2 NCBI reference genome entry NC_045512.2, identical to GenBank entry MN908947.3. The definition of domain boundaries for the IDR1-NTD-IDR2 fragment (1–248) was guided by the SARS-CoV homologue (Chang et al. 2014).

A codon-optimized expression construct of SARS-CoV-2 IDR1-NTD-IDR2 inserted into the pET29b(+) plasmid was obtained from Twist Bioscience.

### Sample preparation

Uniformly ^13^C, ^15^N-labelled IDR1-NTD-IDR2 protein was expressed in *E*. *coli* strain BL21 (DE3). The culture was grown in 1 L LB medium at 37 °C until OD_600_ reached 0.8, then transferred in 250 mL of labelled minimal medium (4x) containing 0.25 g/L ^15^NH_4_Cl (Cambridge Isotope Laboratories), 0.75 g/L [U]^13^C_6_-d-glucose (Eurisotop). After 1 h of metabolite clearance, the culture was induced with 0.2 mM isopropyl-beta-thiogalactopyranoside (IPTG) at 18 °C for 16/18 h.

The cell pellet was resuspended in 25 mM 2-Amino-2-(hydroxymethyl)-1,3-propanediol (TRIS), 1.0 M sodium chloride, 5% glycerol, DNAse, RNAse and 500 µL of 100 × stock of protease inhibitor cocktail (SIGMA) at pH 8.

Cells were disrupted by sonication. The supernatant was cleared by centrifugation (50′, 30,000×*g*, 4 °C), then the cleared supernatant was dialyzed overnight at 4 °C into 25 mM TRIS pH 7.2 (binding buffer).

The protein was purified with ion-exchange chromatography using an HiTrap SP FF 5 mL column and a 70% gradient of 25 mM TRIS, 1 M NaCl pH 7.2. Fractions containing pure protein were pooled and concentrated using 15 mL and 0.5 mL Centricon centrifugal concentrators (MW cutoff 10 kDa).

Final NMR samples were 280 µM IDR1-NTD-IDR2, 25 mM TRIS pH 6.5, 450 mM sodium chloride, 0.02% NaN_3_, 5% (v/v) D_2_O in water.

### NMR experiments

All the NMR experiments were acquired at 298 K. Carbon-13 direct detected NMR experiments were acquired on a 16.4 T Bruker AVANCE NEO spectrometer operating at 700.06 MHz ^1^H, 176.05 MHz ^13^C, and 70.97 MHz ^15^N frequencies, equipped with a 5 mm cryogenically cooled probehead optimized for ^13^C direct detection (TXO). Proton direct detected NMR experiments were acquired on a 28.3 T Bruker AVANCE NEO spectrometer operating at 1200.85 MHz ^1^H, 301.97 MHz ^13^C, and 121.70 MHz ^15^N equipped with a 3 mm cryogenically cooled triple-resonance probehead (TCI).

Backbone assignment was performed by analyzing 2D and 3D ^1^H and ^13^C direct detected experiments. In particular, 2D-[^1^H, ^15^N]-HSQC, 2D-[^1^H, ^15^N]-BEST-TROSY (BT), 2D-CON, 2D-(H)CACO and 2D-(H)CBCACO experiments were performed. Moreover, a series of 3D experiments were acquired: 3D-(H)CBCACON, 3D-(H)CBCANCO, 3D-BT-HNCACB, and 3D-BT-HN(CO)CACB. To compare the resonance values obtained through the carbon detected spectra with the ones obtained with the proton detected ones, 3D-HNCO and 3D-HN(CA)CO were also collected.

All the 2D-^13^C detected experiments were acquired in a version optimized for the detection of the highly flexible regions of the protein (Felli and Pierattelli 2012). Carbon-13 homonuclear decoupling was achieved through the IPAP virtual decoupling approach (Bermel et al. 2006a). 2D-(H)CACO and 2D-(H)CBCACO exploit constant-time evolution in the indirect dimension (Pontoriero et al. 2020). The 2D-CON was acquired both with the ^13^C start variant (Bermel et al. 2006b) as well as with the 2D-(HCA)CON variant (Bermel et al. 2009) to ensure direct detection of proline ^15^N resonances. 3D-(H)CBCACON and 3D-(H)CBCANCO (Bermel et al. 2009) were acquired with high resolution in all detected dimensions. Most relevant acquisition parameters are reported in Table 1.

**Table 1:** Experimental parameters used to collect the NMR experiments

Experiments	Dimension of acquired data			Spectral width (ppm)			NS^a^	d1 + aq (s)^b^	Spectrometer frequency (^1^H) (MHz)
	t1	t2	t3	F1	F2	F3			
^1^H detected									
^1^H-^15^N BEST-TROSY	512 (^15^N)	9676 (^1^H)		41	15		16	0.5	1200
BT-HNCACB	96 (^13^C)	90 (^15^N)	6144 (^1^H)	75	41	14	96	0.2	1200
BT-HN(CO)CACB	96 (^13^C)	80 (^15^N)	6144 (^1^H)	75	41	14	96	0.2	1200
HN(CA)CO	128 (^13^C)	128 (^15^N)	4096 (^1^H)	7	28	18	8	1.0	1200
HNCO	128 (^13^C)	220 (^15^N)	4096 (^1^H)	7	28	18	4	1.0	1200
^13^C detected									
CON	512 (^15^N)	1024 (^13^C)		34	31		32	1.6	700
(HCA)CON	220 (^15^N)	1024 (^13^C)		40	31		16	0.9	700
(H)CACO	330 (^13^C)	1024 (^13^C)		34	30		32	1.0	700
(H)CBCACO	476 (^13^C)	1024 (^13^C)		59	30		32	1.0	700
(H)CBCACON	128 (^13^C)	96 (^15^N)	1024 (^13^C)	58	34	30	4	1.0	700
(H)CBCANCO	96 (^13^C)	96 (^15^N)	1024 (^13^C)	58	34	30	16	1.0	700

^a^Number of acquired scans

^b^Relaxation delay (acquisition time plus recovery delay d1)

Pulse lengths and carrier frequencies generally used for triple resonance experiments were used for the ^13^C detected experiments and are summarized hereafter. The ^1^H carrier was placed at 4.7 ppm for non-selective hard pulses. ^13^C pulses were given at 176.7 ppm, 55.9 ppm, and 45.7 ppm for C′, C^α^ and C^ali^ regions, respectively. ^15^N pulses were given at 124.0 ppm. Q5 and Q3 shapes (Emsley and Bodenhausen 1990) of durations of 300 and 231 μs, respectively, were used for ^13^C band-selective π/2 and π flip angle pulses except for the π pulses that should be band-selective on the C^α^ region (Q3, 900 μs), and for the adiabatic π pulse (Böhlen and Bodenhausen 1993) to invert both C′ and C^α^ (smoothed Chirp 500 μs, 20% smoothing, 80 kHz sweep width, 11.3 kHz RF field strength). Composite pulse decoupling was applied on ^1^H (Waltz-16) (Shaka et al. 1983) and ^15^N (Garp-4) (Shaka et al. 1985) with an RF field strength of 3 kHz and 1 kHz respectively.

^1^H detected experiments, acquired at 1.2 GHz, exploited the BEST-TROSY approach (3D-BT-HNCACB and 3D-BT-HN(CO)CACB) or the sensitivity enhanced approach (3D-HNCO and 3D-HN(CA)CO) for the 3D experiments. The 2D-[^1^H, ^15^N]-BEST-TROSY used sensitivity-enhanced gradient echo/antiecho coherence selection (Czisch and Boelens 1998; Schulte-Herbrüggen and Sørensen 2000) and Band-Selective Excitation Short-Transient (BEST) (Schanda et al. 2006; Lescop et al. 2007; Solyom et al. 2013) approach using exclusively shaped proton pulses. The inter-scan delay was set to 0.2 s. A 2D-[^1^H, ^15^N]-HSQC was also acquired in its fast version which exploits Watergate 3-9-19 pulses for water suppression (Mori et al. 1995). 3D-BT-HNCACB, and 3D-BT-HN(CO)CACB used echo/antiecho gradient selection and semi-constant time in the ^15^N dimension (Schulte-Herbrüggen and Sørensen 2000; Solyom et al. 2013). 3D-HNCO and 3D-HN(CA)CO used sensitivity enhanced approach and selective pulse on the solvent for the water suppression (Kay et al. 1994). C′ and C^α^/C^ß^ selective excitation was exploited through band selective pulses.

Carrier frequencies used for triple resonance experiments in ^1^H detected experiments were the same as for ^13^C detected experiments except for the ^15^N carrier placed at 118.0 ppm. Pulse shapes and lengths for ^13^C band-selective pulses were G4 (Emsley and Bodenhausen 1992) and Q3 (Emsley and Bodenhausen 1990) shapes of durations of 205 and 128 μs, respectively, used for ^13^C band-selective π/2 and π flip angle pulses except for the π pulses that should be band-selective on the C^α^ region (Q3, 525 μs). The ^1^H band-selective pulses on the amide region were Pc9 (Kupce and Freeman 1994) or Eburp2 (Geen and Freeman 1991) for the π/2 and Reburp (Geen and Freeman 1991) or Bip (Smith et al. 2001) for π pulses.

All the spectra were acquired, processed, and analysed by using Bruker TopSpin 4.0.8 software. Chemical shifts were referenced using the ^1^H and ^13^C shifts of DSS. Nitrogen chemical shifts were referenced indirectly using the conversion factor derived from the ratio of NMR frequencies (Markley et al. 1998).

The sequence-specific assignment was performed with the aid of CARA (Keller 2004) and its tool NEASY (Bartels et al. 1995).

### Bioinformatics tools

Several commonly utilized bioinformatics tools were used to predict or evaluate some of the protein features. Peculiarities of the distribution of intrinsic disorder predisposition along the amino acid sequence of the SARS-CoV-2 nucleocapsid protein N were evaluated by several members of the PONDR family (PONDR® VLXT (Romero et al. 2001), PONDR® VL3 (Obradovic et al. 2003), PONDR® VSL2 (Obradovic et al. 2005), and PONDR® FIT (Xue et al. 2010), together with the two versions of IUPred2A designed to predict short and long disordered regions (Mészáros et al. 2018).

The online tool ncSPC available at https://st-protein02.chem.au.dk/ncSPC/ was used to calculate the secondary structure propensity with the obtained assignment (Tamiola and Mulder 2012).

### Assignments and data deposition

**Fig. 2:**
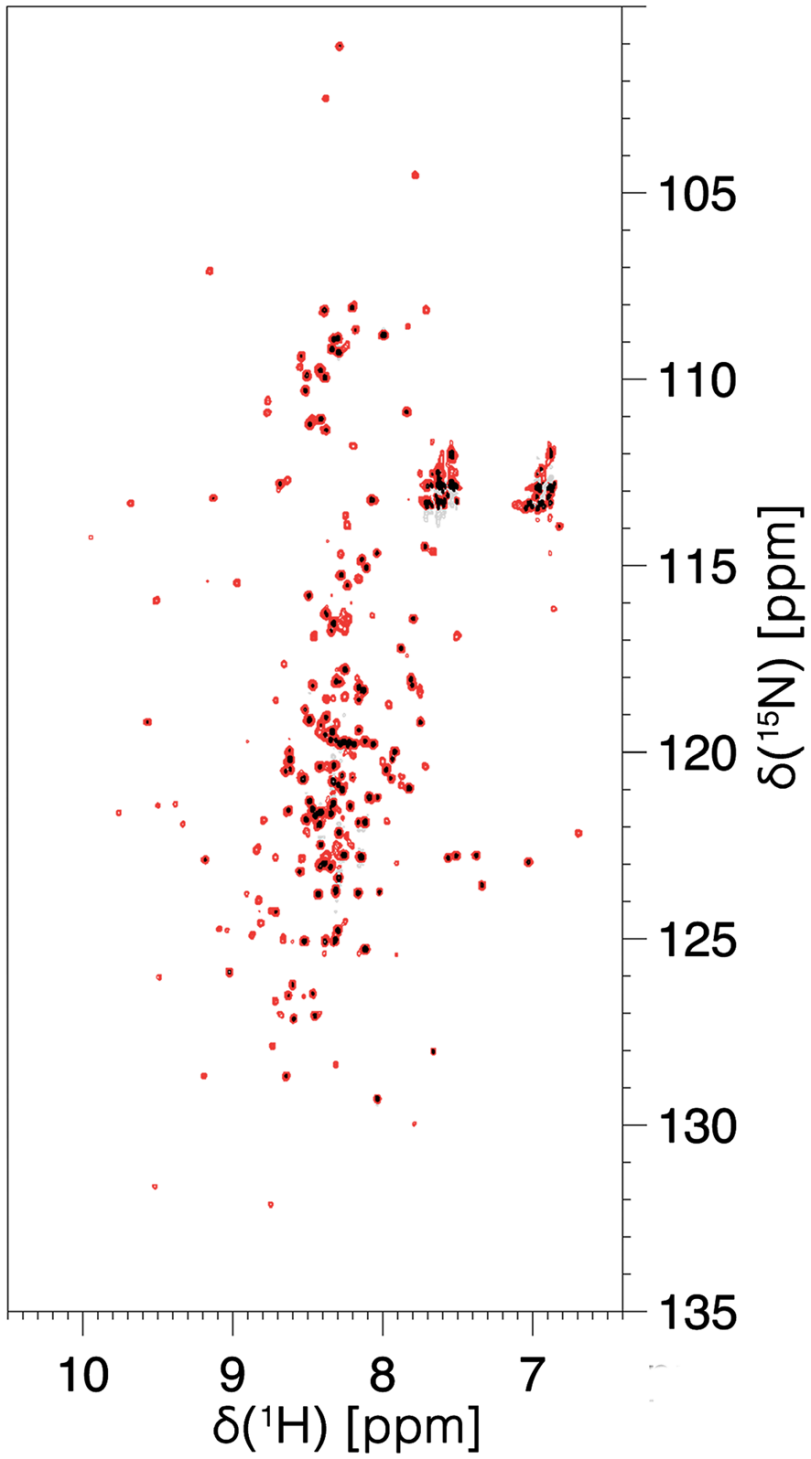
The 2D HN BEST-TROSY of IDR1-NTD-IDR2 construct of the SARS-CoV-2 nucleocapsid protein. The figure shows the superimposition of two different processing of the same spectrum: the black one is optimized for the resolution and the red one is optimized for the signal to noise ratio. The spectrum was collected on a 28.3 T Bruker AVANCE NEO spectrometer operating at 1200.85 MHz ^1^H, 301.97 MHz ^13^C, and 121.70 MHz ^15^N equipped with a 3 mm cryogenically cooled triple-resonance probehead (TCI)

The 2D HN spectrum recorded on the IDR1-NTD-IDR2 (1–248) construct of the SARS-CoV-2 nucleocapsid protein N is shown in Fig. 2. The 2D HN spectrum clearly shows a set of well-resolved NMR signals deriving from the globular NTD domain, as one can verify by superimposing the available sequence-specific assignment (BMRB 34511, Dinesh et al. 2020). In addition, a set of signals, with smaller dispersion and higher intensity, are observed. These are expected to originate from the flexible and disordered fragments of the protein (black contours in Fig. 2).

**Fig. 3:**
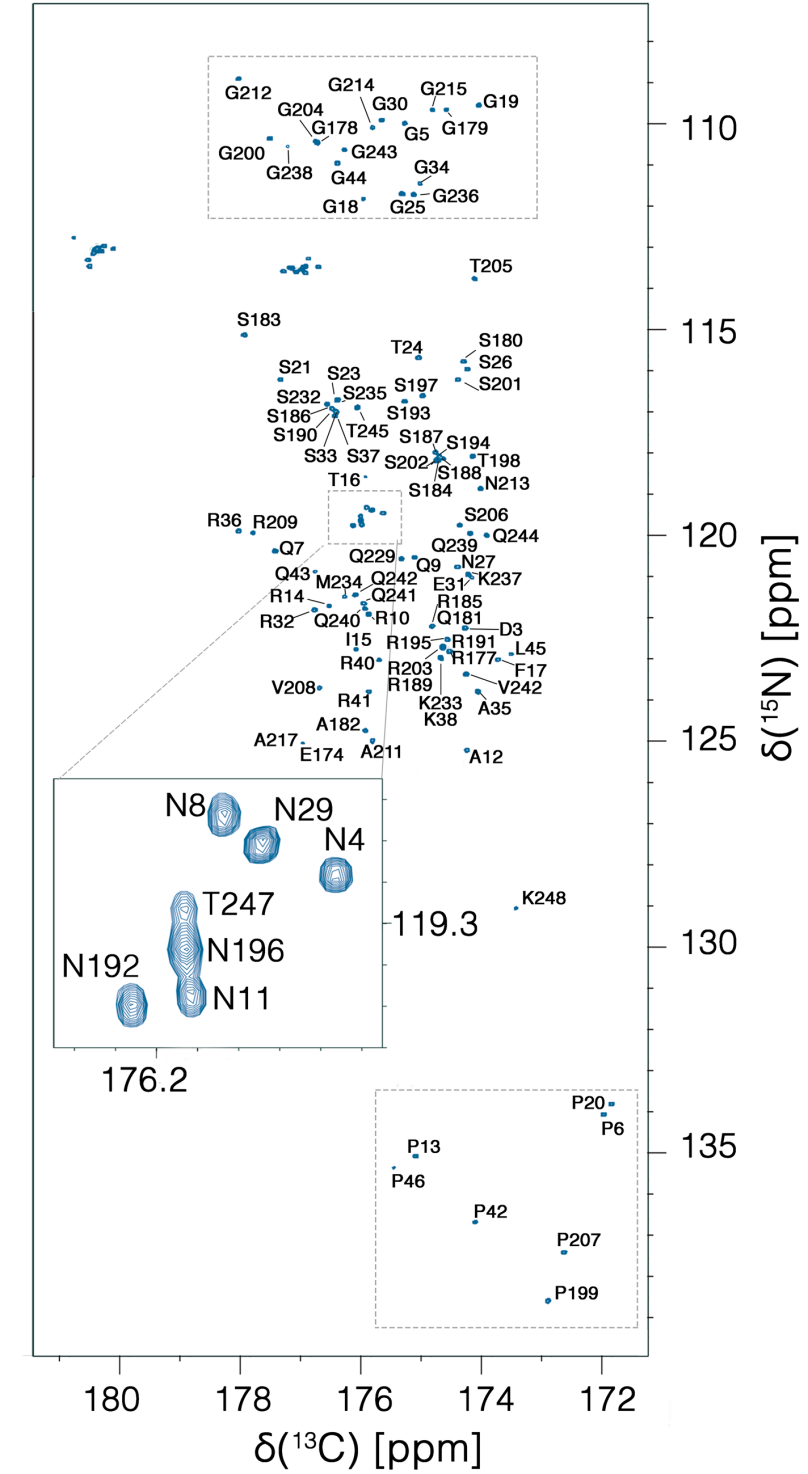
The 2D-CON of IDR1-NTD-IDR2 construct of the SARS-CoV-2 nucleocapsid protein. The high resolution provided by this experiment allows us to easily resolve resonances in the usually very crowded Gly-region (upper squared region) and to directly observe correlations involving proline residues (lower squared region). In the expansion shown in the center of the map the resolution of several repeating fragments comprising asparagine residues can be appreciated (the assignment reported is referred to the amide nitrogen of the mentioned amino acid). The spectrum was acquired on a 16.4 T Bruker AVANCE NEO spectrometer operating at 700.06 MHz ^1^H, 176.05 MHz ^13^C, and 70.97 MHz ^15^N frequencies, equipped with a 5 mm cryogenically cooled probehead optimized for ^13^C direct detection (TXO)

The 2D CON spectrum (Fig. 3) provides information regarding the highly flexible and disordered protein regions. Due to the very different structural and dynamic properties of the globular NTD domain, with the chosen set-up the NMR signals of this region are very weak or absent in the 2D CON. This is exploited to selectively detect the resonances deriving from the two disordered protein regions. Proline residues can be directly monitored through the observation of the C′_i-1_-N_i_ correlations that fall in a very clean region of the CON spectrum (132 < δ(^15^N) < 140 ppm). The observation of only 7 well-resolved cross-peaks in this region (out of 17 expected for this construct) indeed confirms that C′ direct detection selectively picks up the signals of the disordered regions (5 proline residues present in the IDR1 region and 2 in the IDR2 one, Fig. 3 bottom squared region).

Sequence-specific assignment of the resonances can be performed by combining the information available in the 2D ^13^C-detected spectra with that provided by two 3D experiments, the (H)CBCACON and the (H)CBCANCO (Bermel et al. 2009).

**Fig. 4:**
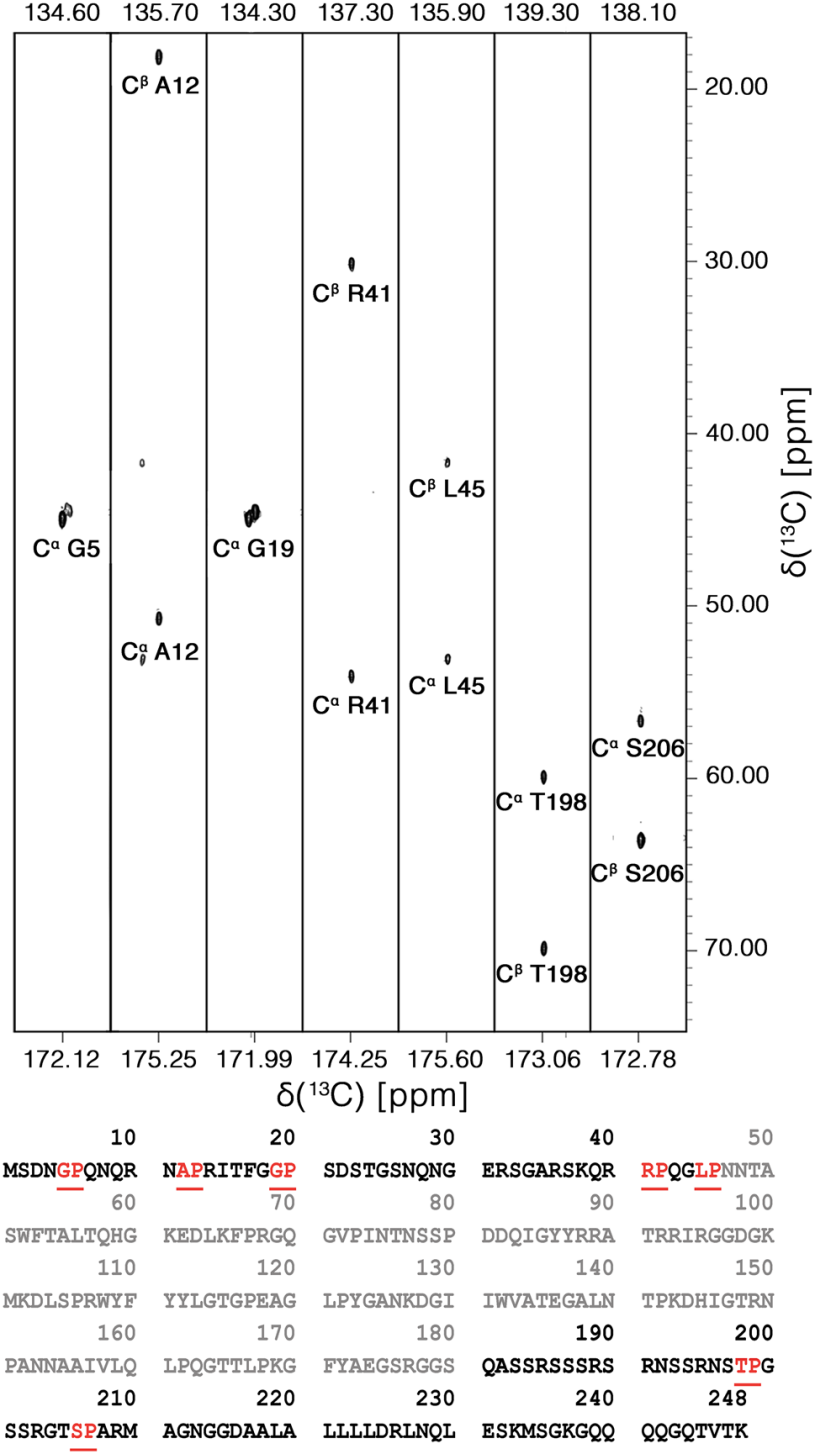
Seven strips derived from the 3D-(H)CBCACON experiment extracted at the ^15^N chemical shift of proline residues. The C′, C^α^ and C^ß^ frequencies belong to the preceding amino acid leading to the X-Pro assignment. The lower part of the figure reports the IDR1-NTD-IDR2 primary sequence in which X-Pro pairs are highlighted. Five proline residues are found in the IDR1 and two in IDR2 domain. The primary sequence of NTD domain is reported in grey. The 3D spectrum was acquired on a 16.4 T Bruker AVANCE NEO spectrometer operating at 700.06 MHz ^1^H, 176.05 MHz ^13^C, and 70.97 MHz ^15^N frequencies, equipped with a 5 mm cryogenically cooled probehead optimized for ^13^C direct detection (TXO)

It is worth noting that proline resonances provide a useful starting point for sequence-specific assignment. The particular ^15^N chemical shift range expected for proline nitrogen signals (N_i_) and the fact that this is correlated to resonances of the preceding amino acid (C′_i-1_, C^α^_i-1_, C^β^_i-1_) through the 2D CON and 3D (H)CBCACON spectra constitute two features that allow us to unambiguously identify the type of dipeptide (X_i-1_-Pro_i_ pair) that gives rise to specific signals as highlighted in Fig. 4. Indeed, the characteristic chemical shifts of C^α^ and C^β^ resonances enable us to recognize glycine, alanine, serine, and threonine residues; the remaining X-Pro pairs can then be easily identified as deriving from leucine and arginine residues by comparison with the primary sequence of the protein. Therefore, already at this very early stage of the sequence-specific assignment process, most of the observed resonances in this region could be assigned to specific amino acids uniquely considering the type of X-Pro pairs present in the intrinsically disordered regions (all resonances could be unambiguously assigned except for the two Gly-Pro pairs). Similarly, inspecting the opposite region of the CON spectrum at low ^15^N chemical shifts (Fig. 3, top squared region) allows us to identify correlations involving ^15^N nuclear spins of glycine residues; correlation to the carbonyl carbon of the previous amino acid (C′_i-1_-N_i_) contributes to an excellent resolution allowing us to count 16 resolved cross peaks in this region in the simple 2D mode. This is in line with the number of glycine residues present in the flexible disordered fragments. The classification of these resonances in X_i-1_-Gly_i_ pairs achieved through inspection of the (H)CBCACON provides further input for their identification, as described above for the case of X_i-1_-Pro_i_ pairs. Complete comparative analysis of the 3D (H)CBCACON and 3D (H)CBCANCO spectra enables the identification of the vast majority of the expected resonances of disordered regions. The excellent resolution obtained in the 2D reference spectra, the CON as well as the (H)CACO and (H)CBCACO, provides valuable support for the analysis of crowded regions of the spectra and to the discrimination between different residue types (Pontoriero et al. 2020).

The information retrieved for the intrinsically disordered regions of the spectra can then be used as a starting point to identify the spin systems also in ^1^H^N^ detected 3D spectra. The latter are much more crowded due to more extensive cross-peak overlap, as well as because the signals of the globular region are also observed. In addition, cross peak intensities are highly heterogeneous due to the different structural and dynamic properties of the globular and disordered domains as well as due to the effects of solvent exchange processes. Therefore, the combined analysis of the two datasets greatly simplifies the identification of the signals deriving from the intrinsically disordered regions. As a further aid to discriminate the different sets of signals, spectra can be processed to enhance resolution, at the expense of signal-to-noise, taking advantage of the long-lived ^15^N coherences of highly flexible regions of the protein as well as exploiting the long FID acquisition times that are possible through the BEST-TROSY approach (Schanda et al. 2006; Lescop et al. 2007; Solyom et al. 2013).

As a result, 98% of the disordered fragment IDR1 (only the first methionine is missing) (BMRB 50619) and 91% of the fragment IDR2 (BMRB 50618) could be assigned in a sequence-specific manner (C′, C^α^, C^β^, N, H^N^) (vide infra). It is interesting to note how the combined use of these complementary datasets (^13^C′- and ^1^H^N^-detected 3D experiments) provides information that is particularly useful to achieve sequence-specific assignment of intrinsically disordered regions also within highly heterogeneous proteins. The set of 2D spectra (HN, CON, (H)CACO, (H)CBCACO), provided they are acquired with high resolution, then becomes a very useful tool to achieve atomic resolution for the vast majority of the amino acids in the highly flexible disordered regions of complex, heterogeneous proteins.

The first two disordered regions of the N protein from SARS-CoV-2 (IDR1 and IDR2) can now be investigated at atomic resolution providing experimental information regarding the many interaction sites that can be predicted through different approaches (Kumar et al. 2008; Giri et al. 2020). The resonances of characteristic amino acids involved in interactions with RNA, such as arginine, serine, glutamine, and glycine residues, which are very abundant in the IDR1 and IDR2 disordered domains, can be detected and most of them can be resolved already in the 2D mode also at physiological pH and temperature conditions. Several signals in low complexity regions, such as the polyQ (238–242) or some repeats located in different positions in the primary sequence (for example the Asn-Arg region reported in the expanded panel in the middle of Fig. 3) can be resolved allowing their high-resolution investigation.

**Fig. 5:**
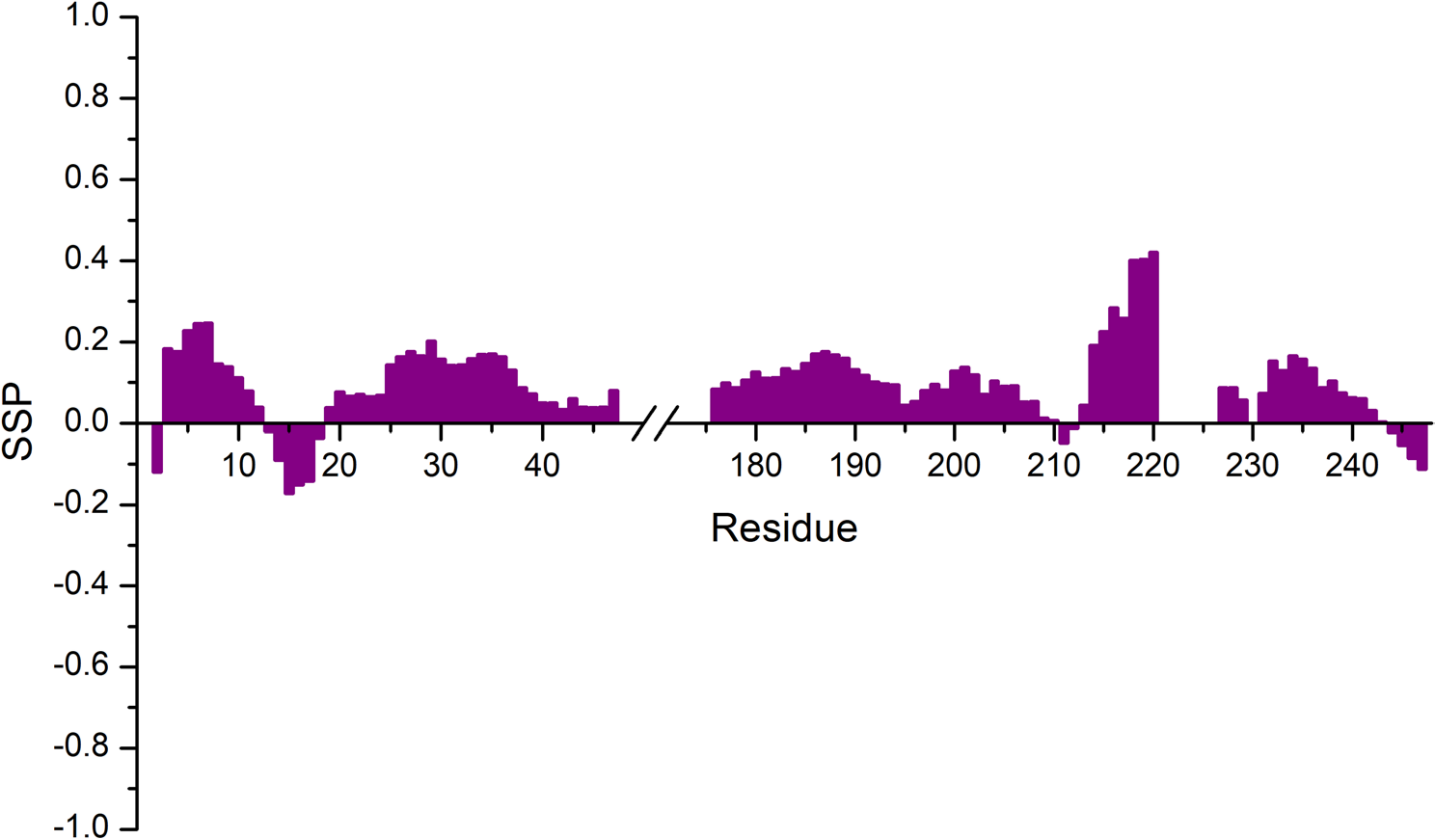
Secondary Structure Propensity (SSP) plot obtained with the assignment reported on the BMRB (50619 and 50618) for the two assigned regions 1–47 and 176–248. Chemical shift values for H^N^, N, C′, C^α^, and C^β^ nuclei were used. The two regions result to be highly disordered with a slight tendency to be in an α-helix conformation for the residues 216–220

Chemical shifts were then used to determine secondary structural propensities as shown in Fig. 5. The data confirm the disordered nature of these fragments, with a moderate propensity to sample a helical conformation in the leucine-rich region (218–232), where few residues (Leu 221, Leu 222, Leu 223, Leu 224, Asp 225, Arg 226, and Leu 230) escaped detection likely because of the signal broadening due to conformational exchange. These experimental results are in agreement with the bioinformatics analysis reported in Fig. 1, which predicts a high extent of disorder for the two IDR regions as well as the presence of some structure in the region 215–232.

The NMR resonance assignments of the IDR1 and IDR2 domains of the N protein from SARS-CoV-2 open the way to understanding the role of these flexible parts of the nucleocapsid protein in modulating its function. The suite of ^13^C detected 2D experiments (CON, (H)CACO, (H)CBCACO) in conjunction with 2D HN correlation experiments provide an excellent tool to monitor at atomic resolution their role in the interactions with RNA, with viral proteins or with proteins of the host, as well as with small molecules as potential drugs, opening the way to radically novel, unexplored approaches in drug discovery.

## Data Availability

The chemical shift values for the ^1^H, ^13^C and ^15^N resonances of the first two flexible linkers of the SARS-CoV-2 nucleoprotein have been deposited in the BioMagResBank (https://www.bmrb.wisc.edu) under accession number 50619 (IDR1, residues 1–47) and 50618 (IDR2, residues 176–248). Spectral raw data (upon request) and assignments are also accessible through https://covid19-nmr.de.
